# Oral Prebiotic Polysaccharide Hydrogels Sustaining Colon Antibody Release Alleviate Inflammatory Bowel Disease

**DOI:** 10.1002/advs.76425

**Published:** 2026-07-06

**Authors:** Xinyi Dong, Huan He, Li Cao, Fenghua Meng, Zhiyuan Zhong

**Affiliations:** ^1^ Biomedical Polymers Laboratory College of Chemistry Chemical Engineering and Materials Science Soochow University Suzhou China; ^2^ College of Pharmaceutical Sciences Soochow University Suzhou China

**Keywords:** antibody delivery, biologic therapy, inflammatory bowel disease, oral delivery, stimuli‐responsive hydrogels

## Abstract

Inflammatory bowel disease (IBD) is a chronic systemic immune disorder with unclear pathogenesis that involves complex interplay between inflammation, gut microbiota, and immune dysregulation. The advance of antibodies targeting tumor necrosis factor‐alpha and interleukin‐12/23, e.g., infliximab (IFX) and ustekinumab (UTK), has greatly improved the clinical treatments. The current intravenous dosing scheme, however, yields partial responses with potentially severe systemic side effects. Here, we report that oral prebiotic polysaccharide hydrogels based on inulin and alginate‐dopamine conjugate (predaGel) mediating efficient and sustained colon antibody delivery and reactive oxygen species (ROS) scavenging effectively alleviate IBD. predaGel with pH and enzyme dual responsiveness is stable in the stomach, adhesive to the intestine, and slowly degrading in the colon, resulting in site‐specific and controlled release of IFX. The mucosal retention, ROS scavenging, and gut microbiota remodeling properties endowed by dopamine and inulin in predaGel not only prevent diarrhea and suppress oxidative stress but also prolong drug function and restore immune homeostasis. Intriguingly, oral administration of IFX and/or UTK‐encapsulated predaGel cures IBD and restores colon function, offering an effective oral alternative to IFX injection. This oral prebiotic hydrogel strategy, with simplicity and multi‐functions, presents a potentially safer and better biologic therapy for IBD.

## Graphical abstract

An orally administered prebiotic polysaccharide hydrogel (predaGel) based on inulin and alginate‐dopamine is stable in the stomach, adheres to the intestine, and degrades slowly in the colon. It enables site‐specific delivery of anti‐TNF‐α and anti‐IL‐12/23 antibodies, scavenges reactive oxygen species, and remodels gut microbiota, offering effective and patient‐friendly treatment for inflammatory bowel disease.

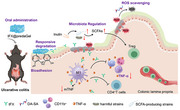

## 1 Introduction

Inflammatory bowel disease (IBD), encompassing Crohn's disease and ulcerative colitis, is a chronic systemic immune disorder driven by complex interactions between genetic susceptibility, environmental factors, gut dysbiosis, and disrupted immune homeostasis, leading to persistent mucosal inflammation and tissue damage [1, 2, 3]. Biologic therapies, particularly monoclonal antibodies targeting tumor necrosis factor‐alpha (TNF‐α) (e.g., infliximab, adalimumab) or interleukin‐12/23 (IL‐12/23) (e.g., ustekinumab), represent a significant advancement over conventional immunosuppressants, improving remission rates, promoting mucosal healing, and reducing surgeries [4, 5, 6]. However, their systemic delivery routes carry substantial limitations: (1) inadequate drug levels at the inflamed colonic mucosa, often resulting in partial or non‐response, and (2) high systemic drug concentration that increases risks such as infections, infusion reactions, and immunogenicity [7, 8, 9].

Oral antibody delivery offers many advantages, including increased mucosal drug exposure and reduced systemic risks [10, 11, 12]. However, this approach faces significant barriers, including gastric and enzymatic degradation, and poor colonic absorption [13, 14]. The past years have witnessed the development of various strategies such as transmucosal‐penetrating chitosan/antibody nanocomplexes, inflammation‐targeted montmorillonite, and edible polysaccharide hydrogels to improve oral antibody delivery [15, 16, 17]. Hydrogels composed of sodium alginate, hyaluronic acid, and chitosan, which are capable of protecting antibodies from enzymatic hydrolysis and, to some extent, prolong intestinal retention, have gained particular interest [18, 19, 20, 21, 22]. The reported hydrogels, typically involving chemical cross‐linking, however, face challenges of antibody deactivation, insufficient bioadhesion, rapid excretion, limited reactive oxygen species (ROS) scavenging, and/or lack of microbiota modulation.

**FIGURE 1:**
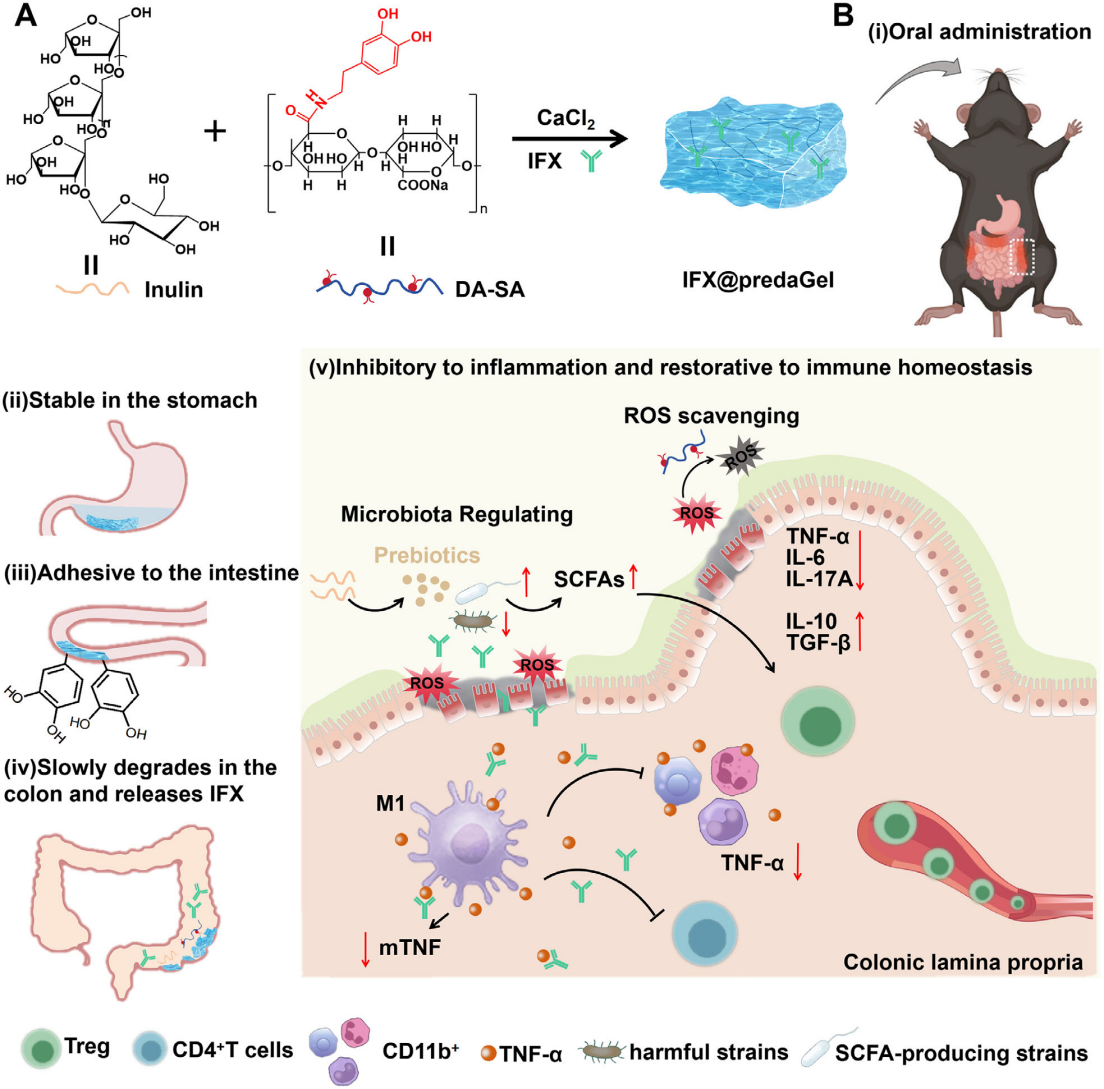
Schematic illustration of IFX@predaGel synthesis (A) and its therapeutic mechanism for IBD treatment (B). (i) IFX@predaGel is given by oral gavage administration, (ii) it is stable in the stomach, (iii) it adheres to the intestinal mucosa via polyphenol‐mediated adhesion, (iv) it degrades in the inflamed colon, and (v) DA‐SA scavenges intestinal ROS, IFX selectively blocks the TNF‐α signaling pathway, and inulin metabolites remodel gut microbiota.

Here, we report that oral prebiotic polysaccharide hydrogels based on inulin and alginate‐dopamine conjugate (predaGel) enable high‐efficacy antibody therapy for IBD (Figure 1). Inulin is only degraded by inulinase in the colon, and more interestingly its metabolites possess prebiotic activity and intestinal flora modulation ability [23, 24, 25, 26], which endows inulin hydrogels with colon‐specific delivery and flora homeostasis restoration functions [27, 28]. The dopamine in predaGel will not only enhance its bioadhesion and drug retention in the inflammation site but also effectively scavenge ROS [29, 30, 31]. Interestingly, our results show that predaGel favors oral delivery and, when loaded with infliximab or infliximab/ustekinumab (IFX/UTK), cures colitis in a mouse model and restores colon function, offering an effective oral alternative to infliximab injection. predaGel with colon‐specific antibody delivery, ROS scavenging, and gut microbiota remodeling properties provides a patient‐friendly, efficient, and potentially safer therapeutic strategy for IBD.

## 2 Results and Discussion

### 2.1 Construction of IFX@predaGel

**FIGURE 2:**
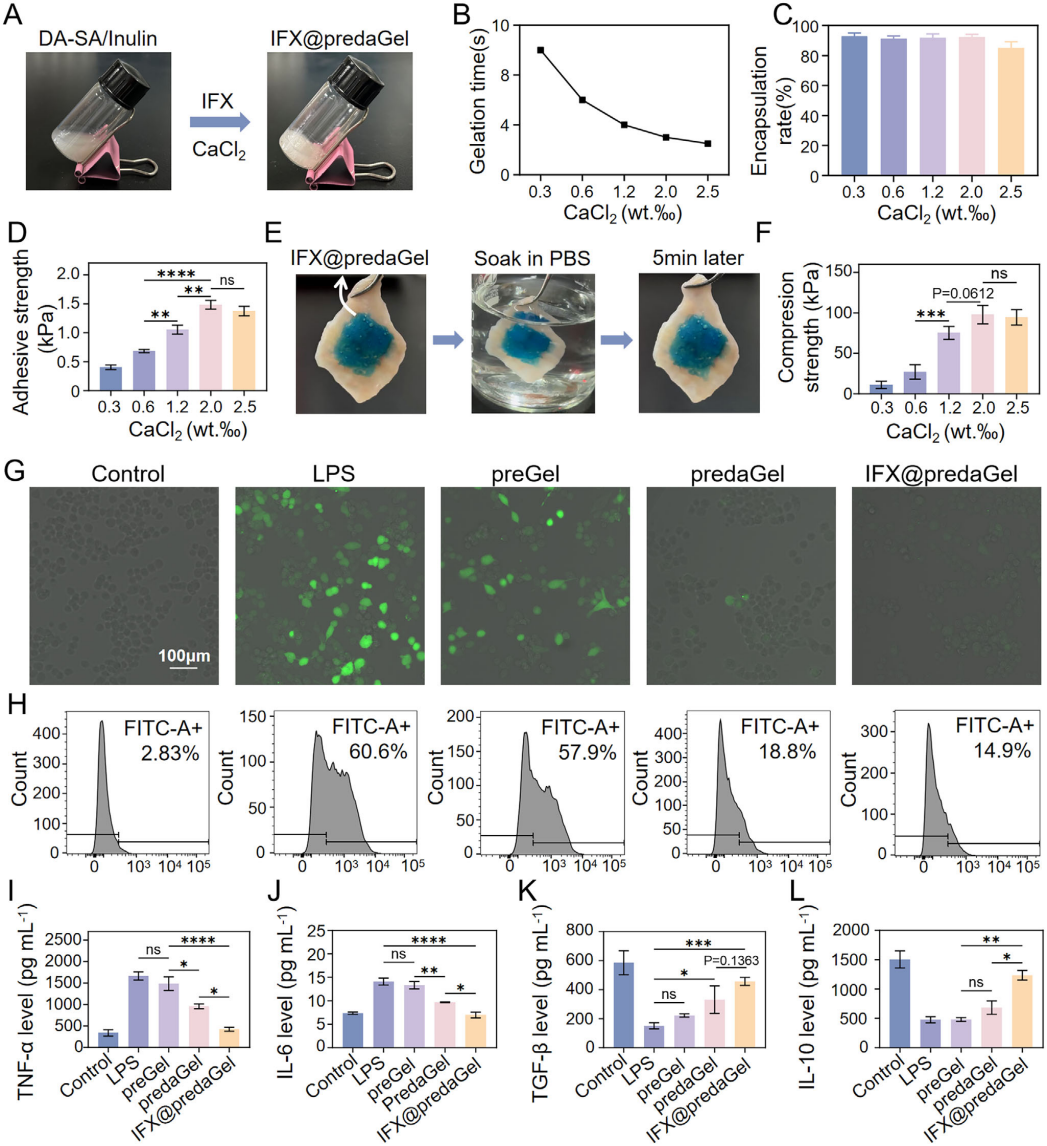
Characterization of IFX@predaGel and its antioxidant and anti‐inflammatory properties. (A) Photographs depicting the self‐curing process of IFX@predaGel. (B) Gelation time of IFX@predaGel at different concentrations of CaCl_2_ (*n* = 3). (C) Encapsulation efficiency of Cy5.5‐IFX in IFX@predaGel (*n* = 3). (D) Adhesion strength of IFX@predaGel with different concentrations of CaCl_2_ on rabbit colon (*n* = 3). (E) Robust adhesion of IFX@predaGel to rabbit colon (*n* = 3). (F) Compressive strength of IFX@predaGel at varying concentrations of CaCl_2_ (*n* = 3). (G) Representative fluorescence images of intracellular ROS staining and (H) corresponding flow cytometry profiles of 2',7'‐dichlorodihydrofluorescein diacetate (DCFH‐DA) labeled RAW264.7 cells subjected to different treatments. (I) Enzyme‑linked immunosorbent assay (ELISA) quantification of extracellular TNF‐α, IL‐6, transforming growth factor‐beta (TGF‐β), and IL‐10 levels (*n* = 3). (Data are expressed as mean ± SD; ^*^ *p* < 0.05, ^**^ *p* < 0.01, ^***^ *p* < 0.001, ^****^ *p* < 0.0001).

IFX@predaGel was fabricated from the physical cross‐linking of inulin and sodium alginate‐dopamine conjugate (DA‐SA). DA‐SA was acquired with a degree of substitution of 12.5% as determined by ultraviolet–visible (UV–vis) spectrophotometry (Figure S1). Inulin and DA‐SA were dissolved at 70°C and cooled to room temperature, to which infliximab (IFX) and calcium chloride (CaCl_2_) were added and allowed to stand for 12 h. Ca^2^^+^ rapidly induced gelation (Figure 2A), with a gelation time of 3–10 s depending on CaCl_2_ concentration (Figure 2B). At 0.3–2.0 wt.‰ CaCl_2_, IFX encapsulation efficiency approached 95% (Figure 2C). IFX@predaGel at 2.0 wt.‰ CaCl_2_ exhibited excellent syringeability (Figure S2) and exceptional tissue adhesion (Figure 2D), attributed primarily to the catechol groups of dopamine, which can form both non‐covalent (e.g., hydrogen bonding) and covalent (e.g., Michael addition with tissue surface amines) interactions with tissues [29, 30], enabling strong adhesion to various substrates including vertically suspended rabbit colon tissue immersed in phosphate‑buffered saline (PBS) (Figure 2E and Figures S3 and S4). IFX@predaGel at 2.0 wt.‰ CaCl_2_ also displayed the best compressive strength (Figure 2F and Figure S5) and anti‐swelling properties (Figure S6).

The composition of the hydrogel, specifically the inulin‐to‐DA‐SA mass ratio, was found to critically influence its properties. As this ratio increased from 0:1 to 8:1, the hydrogel demonstrated progressively enhanced mechanical strength (Figure S7) and improved anti‐swelling behavior (Figure S8). This enhancement is likely attributable to the increased formation of hydrogen bonds between the abundant hydroxyl groups of inulin and DA in DA‐SA, which complements the ionic cross‐linking by Ca^2^^+^ and creates a denser network, as commonly observed in other polysaccharide‐based composite hydrogels [32, 33]. However, when the ratio exceeded 8:1, excessive self‐association among inulin molecules predominated, which impeded effective Ca^2^^+^‐mediated cross‐linking with DA‐SA, resulting in a decline in gel performance. Notably, pure inulin gel failed to maintain structural integrity and disintegrated within 5 min in PBS (Figure S9), rendering it unsuitable as a standalone drug carrier. Therefore, in subsequent studies, IFX@predaGel with an inulin‐to‐DA‐SA mass ratio of 8:1 and a CaCl_2_ concentration of 2.0 wt.‰ was employed.

The drug release profile showed that IFX@predaGel sustained IFX release over 22 h, which was significantly longer than that of control gels based solely on Ca^2^^+^/DA‐SA (10 h) (Figure S10). The prolonged release kinetics of IFX@predaGel align with the robust, low swelling rate observed in the mechanical tests and suggest a diffusion‐controlled release mechanism, which is highly desirable for long‐term drug delivery applications [34]. The biological activity of the released IFX remained intact, as it effectively neutralized extracellular TNF‐α in lipopolysaccharide (LPS)‐stimulated macrophages after 24 h of co‐incubation with IFX@predaGel (Figure S11), demonstrating that the fabrication process preserves the therapeutic protein's structure and function. Furthermore, IFX@predaGel exhibited excellent long‐term storage stability, as evidenced by the unchanged morphology, encapsulation efficiency, and IFX bioactivity after 14 days of storage at 4°C (Figure S12).

The incorporation of dopamine endowed the hydrogel with additional pharmacological benefits. predaGel effectively scavenged 2,2‐Diphenyl‐1‐picrylhydrazyl (DPPH) radicals, whereas a dopamine‐free preGel control showed no scavenging activity against DPPH radicals under identical conditions (Figure S13). This confirms that the observed antioxidant activity is intrinsically linked to the catechol groups of dopamine, consistent with previous reports on dopamine‐modified biomaterials [35]. The ROS‐scavenging capacity of predaGel was further validated in a biologically relevant LPS‐stimulated macrophage model (Figure 2G,H). IFX@predaGel significantly reduced intracellular ROS levels, while the preGel control had little effect, underscoring the essential role of the dopamine component in mitigating oxidative stress within an inflammatory microenvironment.

The immunomodulatory effects were subsequently assessed by measuring key macrophage polarization markers. IFX@predaGel significantly downregulated the expression of pro‐inflammatory cytokines (TNF‐α and IL‐6) compared to the preGel control (Figure 2I,J). We attribute this potent anti‐inflammatory effect to a synergistic action between the continuous neutralization of TNF‐α by released IFX and the scavenging of ROS by dopamine, which together disrupt key signaling pathways (e.g., NF‐κB) that drive M1macrophage pro‐inflammatory polarization [36, 37]. Notably, IFX@predaGel simultaneously upregulated the expression of anti‐inflammatory cytokines (TGF‐β and IL‐10) (Figure 2K,L), demonstrating a unique ability not only to suppress the M1 macrophage phenotype but also to promote the M2 macrophage activation state, which is crucial for inflammation resolution and tissue repair. Finally, cytotoxicity assessments confirmed the excellent biocompatibility of IFX@predaGel across a range of IFX concentrations (0.5 to 2.0 wt.‰), supporting its potential for safe in vivo application (Figure S14).

### 2.2 Gastrointestinal Stability and Microbiota‐Responsive Degradation

**FIGURE 3:**
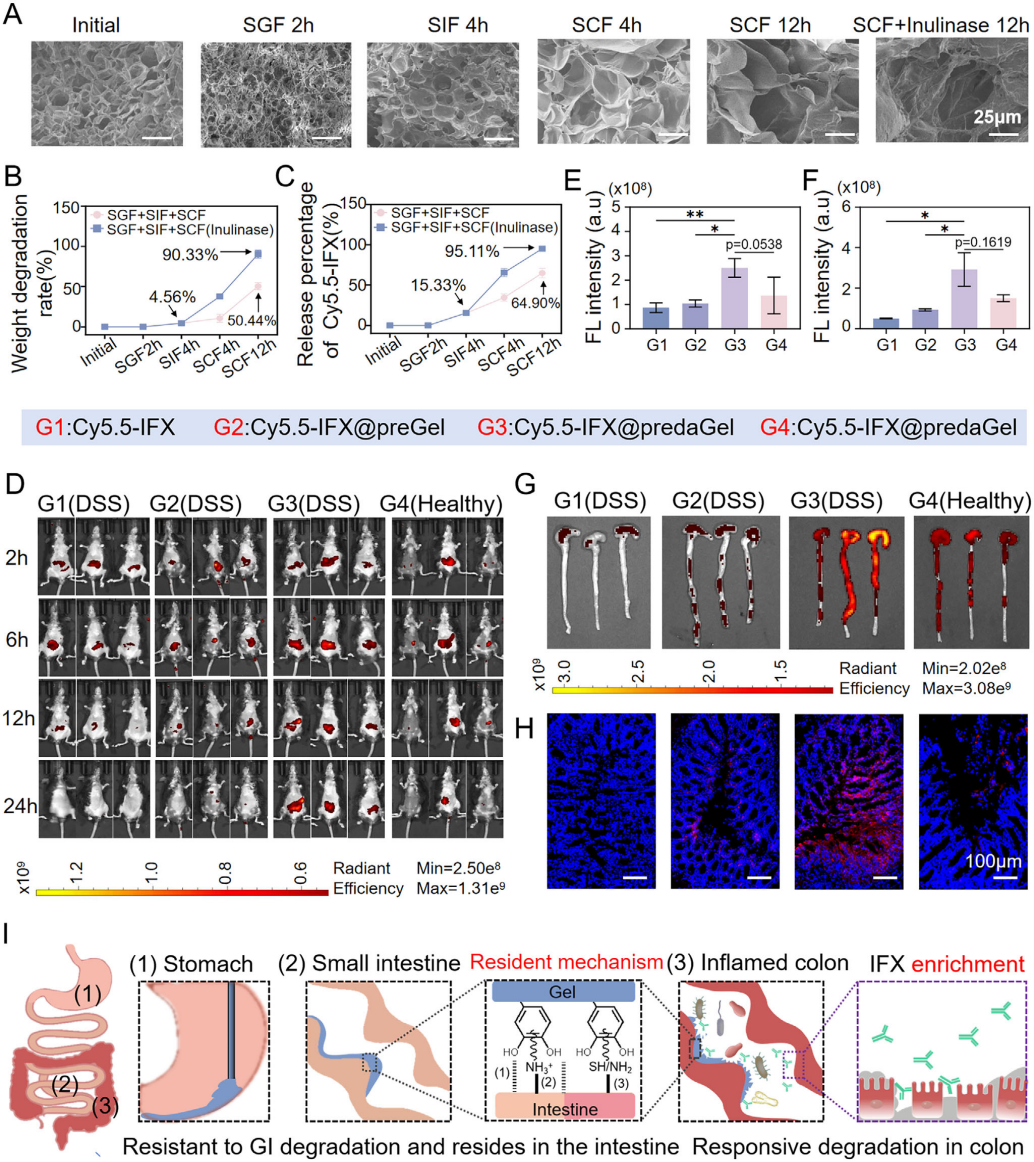
IFX@predaGel exhibits gastric stability, prolonged intestinal retention, and microbiota‐responsive colonic degradation. (A) SEM images of IFX@predaGel after incubation in simulated gastrointestinal fluids at the indicated time points. (B, C) Weight retention of IFX@predaGel and cumulative Cy5.5‐IFX release after 2 h in SGF, 4 h in SIF, 4 and 12 h in SCF (SGF+SIF+SCF), or after 2 h in SGF, 4 h in SIF, 4 h and 12 h in SCF plus inulinase (SGF+SIF+SCF(Inulinase)) (*n* = 3). (D, E) In vivo fluorescence imaging (2/6/12/24 h) and 24 h quantitative analysis in healthy and DSS‐induced colitis mice following oral gavage of Cy5.5‐IFX, Cy5.5‐IFX@preGel, or Cy5.5‐IFX@predaGel (*n* = 3). (F–H) Ex vivo colon fluorescence imaging (F), quantification (G), and cryosections (H; 4′,6‑diamidino‑2‑phenylindole (DAPI) nuclear counterstain) at 24 h post‐gavage (same treatment groups as D, E) (*n* = 3). (I) The mechanism of IFX@predaGel involves gastric stability, intestinal retention, and microbiota‐triggered colonic degradation. This enables targeted IFX accumulation within inflamed tissues, where it penetrates the mucosal epithelium and lamina propria to neutralize TNF‐α. (Data are expressed as mean ± SD; ^*^ *p* < 0.05, ^**^ *p* < 0.01).

IFX would degrade rapidly in gastric juice [38]. To evaluate its stability, Cy5.5‐labeled IFX‐loaded predaGel (Cy5.5‐IFX@predaGel) was prepared and sequentially exposed to simulated gastric fluid (SGF) for 2 h and simulated intestinal fluid (SIF) for 4 h. Scanning electron microscopy (SEM) analysis revealed that exposure to SGF reduced gel volume and pore size (Figure 3A and Figure S15), a phenomenon resulting from protonation‐induced charge neutralization and osmotic dehydration, and no significant degradation or release of Cy5.5‐IFX was detected (Figure 3B,C). Real‐time monitoring using an in vitro simulated human gastric system [39] confirmed that the gel remained structurally intact after 2 h in the stomach (Figure S16). Following 4 h in SIF, the gel surface exhibited slight dissolution accompanied by modest pore enlargement (Figure 3A), resulting in approximately 4.56% degradation and 15.33% antibody release (Figure 3B,C). The antibody retention capacity of predaGel in the stomach and small intestine is superior to that reported for Alg‐AEMA hydrogel microcapsules [15]. Importantly, IFX@predaGel extracted after SGF/SIF exposure effectively neutralized extracellular TNF‐α expression during a 24 h co‐incubation with LPS‐stimulated macrophages (Figure S17). Moreover, after SGF (2 h), SIF (4 h), and then simulated colon fluid (SCF, 4 h) incubation, the gel still exhibited good adhesion strength to the rabbit colon, demonstrating its adhesion stability in gastrointestinal fluids (Figure S18). Collectively, these results demonstrate that IFX@predaGel resists degradation and preserves the bioactivity of IFX throughout the upper gastrointestinal tract.

The colon‐responsive degradation and IFX release kinetics of Cy5.5‐IFX@predaGel were further evaluated using SCF, either with or without inulinase. SEM analysis indicated gel swelling and increased pore size after 4 or 12 h in SCF alone (Figure 3A). In contrast, incubation in inulinase‐supplemented SCF caused structural collapse, resulting in a significantly higher total mass loss (90.33% after 12 h) compared to SCF without inulinase (50.44%) (Figure 3B). Correspondingly, the Cy5.5‐IFX release rate reached 64.90% in plain SCF and 95.11% in inulinase‐supplemented SCF after 12 h (Figure 3C). These findings indicate that IFX@predaGel, while stable in the stomach and small intestine, is subject to colon‐specific degradation and antibody release.

### 2.3 Targeted IFX Delivery to the Inflamed Colon

We evaluated IFX delivery efficiency in a mild colitis mouse model induced by 4‐day administration of 3% (w/v) dextran sulfate sodium (DSS). In vivo fluorescence imaging at 2, 6, 12, and 24 h post‐oral gavage revealed distinct biodistribution patterns. The Cy5.5‐IFX group exhibited transient abdominal fluorescence due to gastrointestinal degradation (Figure 3D,E), while Cy5.5‐IFX@predaGel showed persistent abdominal signals. Quantitative analysis demonstrated significantly higher fluorescence intensities in both abdominal regions and excised colons at 24 h for Cy5.5‐IFX@predaGel than Cy5.5‐IFX@preGel (Figure 3F,G). This enhanced retention stems from predaGel's gastrointestinal stability and prolonged intestinal residence mediated by mussel‐inspired catechol adhesion. Notably, DSS‐treated mice receiving Cy5.5‐IFX@predaGel displayed stronger abdominal and colon signals than healthy counterparts. Colon cryosections confirmed intensified red fluorescence specifically localized to ulcerated mucosa in the Cy5.5‐IFX@predaGel group (Figure 3H), indicating IFX released in the colon can easily penetrate the lesion and effectively neutralize the overexpressed TNF‐α. In healthy colons, intact mucosa restricts antibody penetration, leading to peristaltic clearance. Collectively, predaGel can resist gastric acidity, prolong intestinal retention via mussel‐mimetic catechol adhesion, and undergo microbiome‐triggered degradation in the colon, enabling targeted accumulation of IFX within inflamed tissues (Figure 3I).

### 2.4 Oral IFX@predaGel Effectively Treats DSS‐Induced Colitis Mouse Model

**FIGURE 4:**
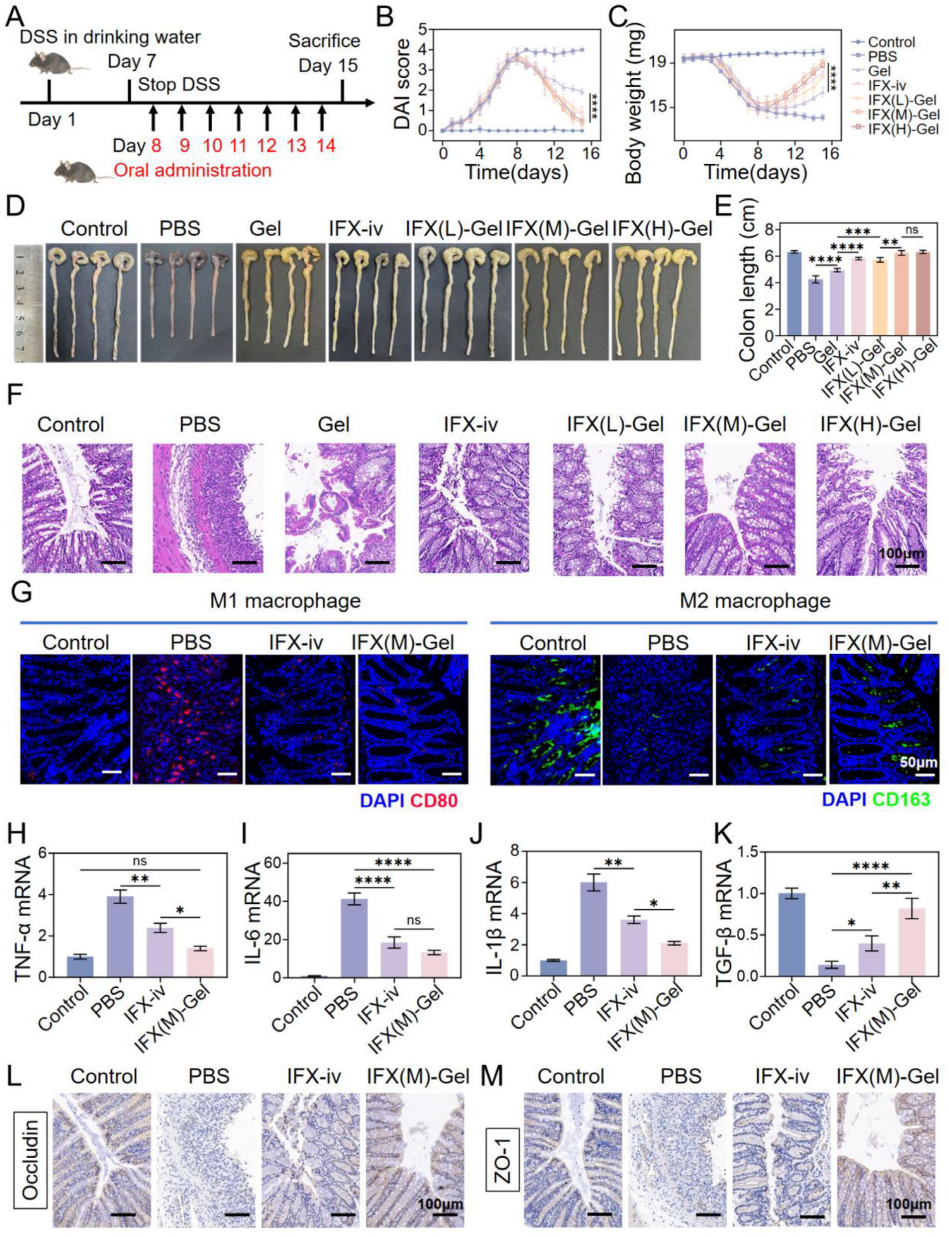
Therapeutic efficacy of IFX@predaGel in a DSS‐induced colitis mouse model. (A) Schematic of the experimental design. Colitis was induced in mice by providing 3% DSS in sterile drinking water over 7 days. Treatments were administered orally beginning on day 8, and all mice were euthanized on day 15. (B) Disease activity index (DAI) scores and (C) body weight changes (*n* = 4). (D) Representative images and (E) colon length measurements (*n* = 4). (F) Representative hematoxylin and eosin (H&E)‐stained micrographs of colonic tissue sections. (G) Immunofluorescence analysis of macrophage polarization in colon tissues: M1 macrophages (CD80, red) and M2 macrophages (CD163, green); nuclei counterstained with DAPI (blue). (H–K) Relative mRNA expression levels of colonic inflammatory cytokines (*n* = 3). (L) Immunohistochemical staining of tight junction protein occludin and (M) Zonula occludens‐1 (ZO‐1) in colon tissues. (Data are expressed as mean ± SD; ^*^ *p* < 0.05, ^**^ *p* < 0.01, ^***^ *p* < 0.001, ^****^ *p* < 0.0001).

The efficacy of IFX@predaGel was studied in DSS‐induced acute colitis, which was established by feeding mice with 3% DSS drinking water ad libitum for 7 days. Treatments commenced on day 8 and comprised the following groups: PBS, predaGel alone (Gel), IFX@predaGel loaded with low (5 mg kg^−1^), medium (10 mg kg^−1^), or high‐dose (20 mg kg^−1^) IFX (denoted as IFX(L)‐Gel, IFX(M)‐Gel, and IFX(H)‐Gel, respectively), and a group receiving intravenous injection of IFX (5 mg kg^−1^, IFX‐iv). All formulations were administered in 200 µL. All mice were sacrificed on day 15 for analysis (Figure 4A). All treatment groups exhibited potent anti‐inflammatory effects, evidenced through alleviated fecal bleeding (Figure S19), reduced disease activity index scores (Figure 4B), mitigated body weight loss (Figure 4C), restored colon length (Figure 4D,E), and normalized spleen weight/size (Figure S20). Histopathological analysis demonstrated superior protection by IFX(M)‐Gel and IFX(H)‐Gel against DSS‐induced damage, including crypt erosion, epithelial damage, and inflammatory cell infiltration (Figure 4F). Blinded histopathological scoring confirmed significantly lower inflammation scores in these two groups (Figure S21). The mild anti‐inflammatory effect in the Gel group likely stems from its ROS scavenging effect. IFX(L)‐Gel treatment demonstrated efficacy comparable to IFX‐iv, confirming successful IFX delivery to inflamed colon tissue and subsequent TNF‐α neutralization. Crucially, IFX(M)‐Gel achieved optimal therapeutic efficacy equivalent to the higher‐dose IFX(H)‐Gel group, indicating that a moderate IFX dose sufficiently neutralizes TNF‐α in inflamed colon tissue. IFX(M)‐Gel was selected for subsequent mechanistic investigations.

Immunofluorescence staining demonstrated that IFX@predaGel significantly reduced pro‐inflammatory M1 macrophages infiltration while markedly increasing anti‐inflammatory M2 macrophages compared to IFX‐iv (Figure 4G). Reverse transcription quantitative polymerase chain reaction (RT‐qPCR) analysis confirmed potent suppression of pro‐inflammatory cytokines (IL‐6, IL‐1β, TNF‐α) and enhanced expression of the reparative cytokine TGF‐β in colon tissue (Figure 4H–K). IFX@predaGel significantly upregulated tight junction proteins (occludin, ZO‐1) (Figure 4L,M), indicating restored intestinal barrier integrity, and achieved significantly greater reduction in colonic myeloperoxidase (MPO) activity than IFX‐iv (Figure S22), reflecting diminished neutrophil infiltration.

The enhanced anti‐inflammatory and tissue‐repair capacity of oral IFX@predaGel within inflamed colon tissue likely results from (i) reprogrammed macrophage polarization (M1 reduction/M2 increase), (ii) rebalanced cytokine milieu (pro‐inflammatory suppression/TGF‐β promotion), (iii) restored epithelial barrier function (occludin/ZO‐1 upregulation), and (iv) attenuated neutrophil infiltration (MPO activity reduction).

### 2.5 Regulation of Gut Microbiota by IFX@predaGel

**FIGURE 5:**
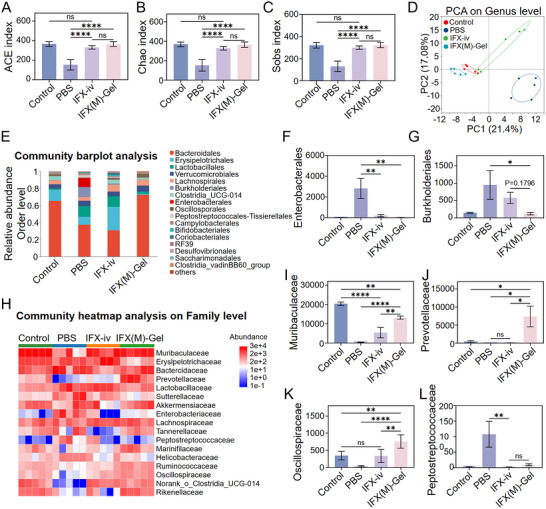
Gut microbiota modulation following IFX@predaGel treatment. (A) ACE index, (B) Chao index, and (C) Sobs index reflecting α‐diversity of the microbial community based on observed operational taxonomic units (*n* = 5). (D) β‐diversity of the gut microbiome visualized by principal coordinates analysis (PCA) (*n* = 5). (E) Histogram of microbial community composition at the order level (*n* = 5). (F) Relative abundance of Enterobacterales and (G) Burkholderiales derived from (E) (*n* = 5). (H) Heatmap of microbial community composition at the family level (*n* = 5). (I) Relative levels of Muribaculaceae, (J) Prevotellaceae, (K) Oscillospiraceae, and (L) Peptostreptococcaceae derived from (H) (*n* = 5). (Data are expressed as mean ± SD; ^*^ *p* < 0.05, ^**^ *p* < 0.01, ^****^ *p* < 0.0001).

To investigate whether IFX@predaGel reshapes the gut microbiota in colitic mice, we performed 16S rRNA gene sequencing. Alpha diversity indices (ACE, Chao, and Sobs) indicated that intravenous IFX (IFX‐iv) and oral IFX@predaGel both significantly increased microbial richness and diversity compared with the PBS group, restoring levels to those approaching healthy controls (Figure 5A–C). Principal component analysis (PCA) of beta diversity revealed that both treatments shifted the overall microbial composition closer to that of the healthy group, although the IFX‐iv group exhibited greater inter‐individual variation (Figure 5D). Community composition analysis showed that the PBS group had significantly elevated abundances of inflammation‐associated bacterial orders (e.g., *Desulfovibrionales*, *Burkholderiales*, and *Enterobacterales*) [40, 41, 42]. Notably, IFX@predaGel treatment reduced these pathogenic orders to near‐healthy levels, with abundances significantly lower than those in the IFX‐iv group (Figure 5E–G and Figure S23). Studies have reported that short‑chain fatty acids (SCFAs) produced by gut flora help to alleviate intestinal inflammation, protect intestinal energy metabolism, and maintain intestinal mucosal integrity and systemic immune homeostasis [43]. Species abundance heatmap analysis (Figure 5H–L and Figure S24) revealed that compared to the IFX‐iv group, IFX@predaGel treatment significantly enriched SCFA‐producing probiotics (e.g., *Muribaculaceae*, *Prevotellaceae*, *Oscillospiraceae*, and *Ruminococcaceae*) [44, 45], while substantially reducing harmful bacteria such as *Peptostreptococcaceae* (associated with autoimmune diseases) [46]. These results suggest that IFX@predaGel treatment not only effectively restored gut microbiota diversity but also reshaped a healthy gut microecology by increasing beneficial bacteria (particularly SCFA‐producing bacteria) and reducing harmful bacteria, which may further modulate the immune system.

### 2.6 IFX@predaGel Maintains Immune Homeostasis and Reduces System Side Effects

**FIGURE 6:**
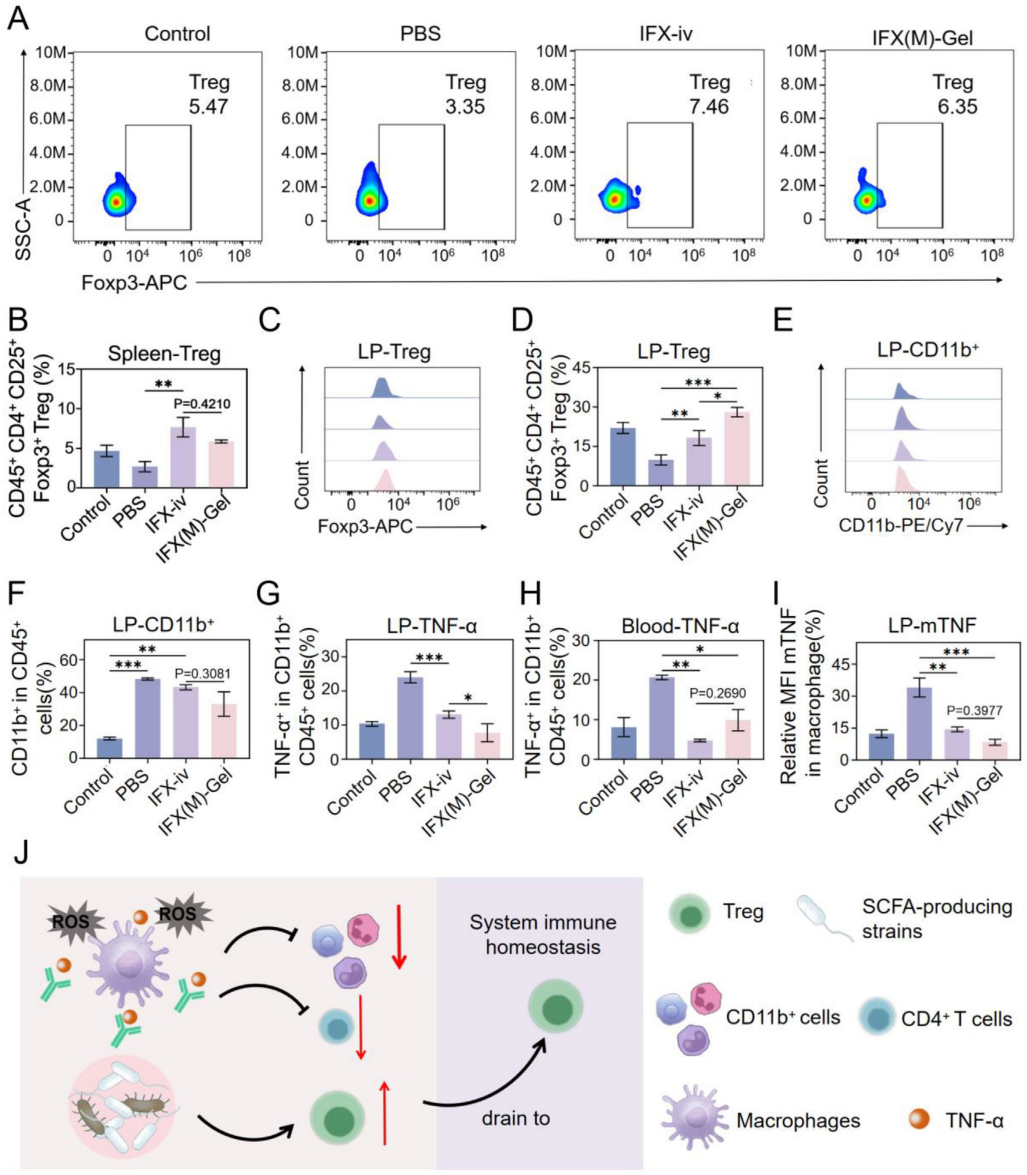
Oral administration of IFX@predaGel maintains immune homeostasis and reduces systemic side effects in colitis mice. (A) Representative flow cytometry plots showing Treg cells (CD45^+^CD4^+^CD25^+^Foxp3^+^) in the spleen. (B) Quantification of splenic Treg cell percentages (*n* = 3). (C) Representative flow cytometry plots and (D) corresponding percentages of Treg cells in the colonic lamina propria (LP) (*n* = 3). (E) Representative flow cytometry plots and (F) corresponding percentages of CD11b^+^ cells in CD45^+^ cells in colonic LP (*n* = 3). (G) The percentage of TNF‐α in CD11b^+^ cells in colonic LP and (H) blood (*n* = 3). (I) The percentage of mTNF in CD11b^+^ cells in mesenteric lymph nodes (*n* = 3). (J) Schematic illustration depicting the regulation of local and systemic immune homeostasis following oral administration of IFX@predaGel in mice with colitis. (Data are expressed as mean ± SD; ^*^ *p* < 0.05, ^**^ *p* < 0.01, ^***^ *p* < 0.001).

To evaluate the impact of orally administered IFX@predaGel on local colonic inflammation and systemic immunosuppression, we analyzed immune cell populations using flow cytometry. Regulatory T cells (Tregs), a subset of CD4^+^ T cells, play a critical role in suppressing excessive immune responses [47]. Compared to the PBS group, both the IFX@predaGel and IFX‐iv groups showed significantly increased proportions of Tregs in the peripheral blood, spleen, and colonic lamina propria (LP) (Figure 6A–D and Figure S25). However, the IFX@predaGel group exhibited a significantly higher proportion of Tregs in the colonic LP compared to the IFX‐iv group, while displaying lower Treg proportions in the peripheral blood and spleen. This distribution pattern suggests that IFX@predaGel therapy promotes local immune homeostasis within the colon while minimizing systemic immunosuppression. Furthermore, IFX@predaGel significantly reduced the total number of colonic CD4^+^ T cells (Figure S26) and suppressed local pro‐inflammatory activity.

Assessment of inflammatory cell infiltration, a correlate of colitis severity [48], using CD11b (a marker for neutrophils, macrophages, and monocytes), revealed that IFX@predaGel more potently suppressed colon CD11b^+^ cell infiltration than IFX‐iv (Figure 6E,F). TNF‐α, a key pro‐inflammatory mediator elevated in IBD and primarily produced by CD11b^+^ cells [49], showed inhibited secretion by colonic LP CD11b^+^ cells after IFX@predaGel treatment (Figure 6G). In contrast, ELISA quantification of serum TNF‐α revealed significantly lower levels in the IFX‐iv group than in the IFX@predaGel group, although flow cytometric analysis of blood showed no significant difference in TNF‐α levels between these groups (Figure 6H and Figures S27 and S28A). Both treatments significantly suppressed serum IL‐6 levels (Figure S28B).

IFX@predaGel, while effectively neutralized membrane‐bound TNF (mTNF) on colonic macrophages (Figure 6I and Figure S29A), demonstrated weaker peripheral blood TNF‐neutralizing capacity than IFX‐iv (Figure S29B,C). This localized action would greatly reduce systemic side effect risks linked to excessive peripheral TNF‐α neutralization [7]. Additionally, IFX@predaGel significantly downregulated T helper 17 (Th17) cell proportions (pro‐inflammatory helper T cells secreting IL‐17A [50]) in mesenteric lymph nodes compared to IFX‐iv (Figure S29D,E). H&E staining of major organs confirmed no systemic toxicity of IFX@predaGel (Figure S30). The above results suggest that IFX@predaGel exerts immunosuppressive effects locally at the inflamed site while preserving systemic immune homeostasis and minimizing peripheral immunosuppression (Figure 6J).

### 2.7 predaGel for Oral Delivery of UTK and IFX/UTK

**FIGURE 7:**
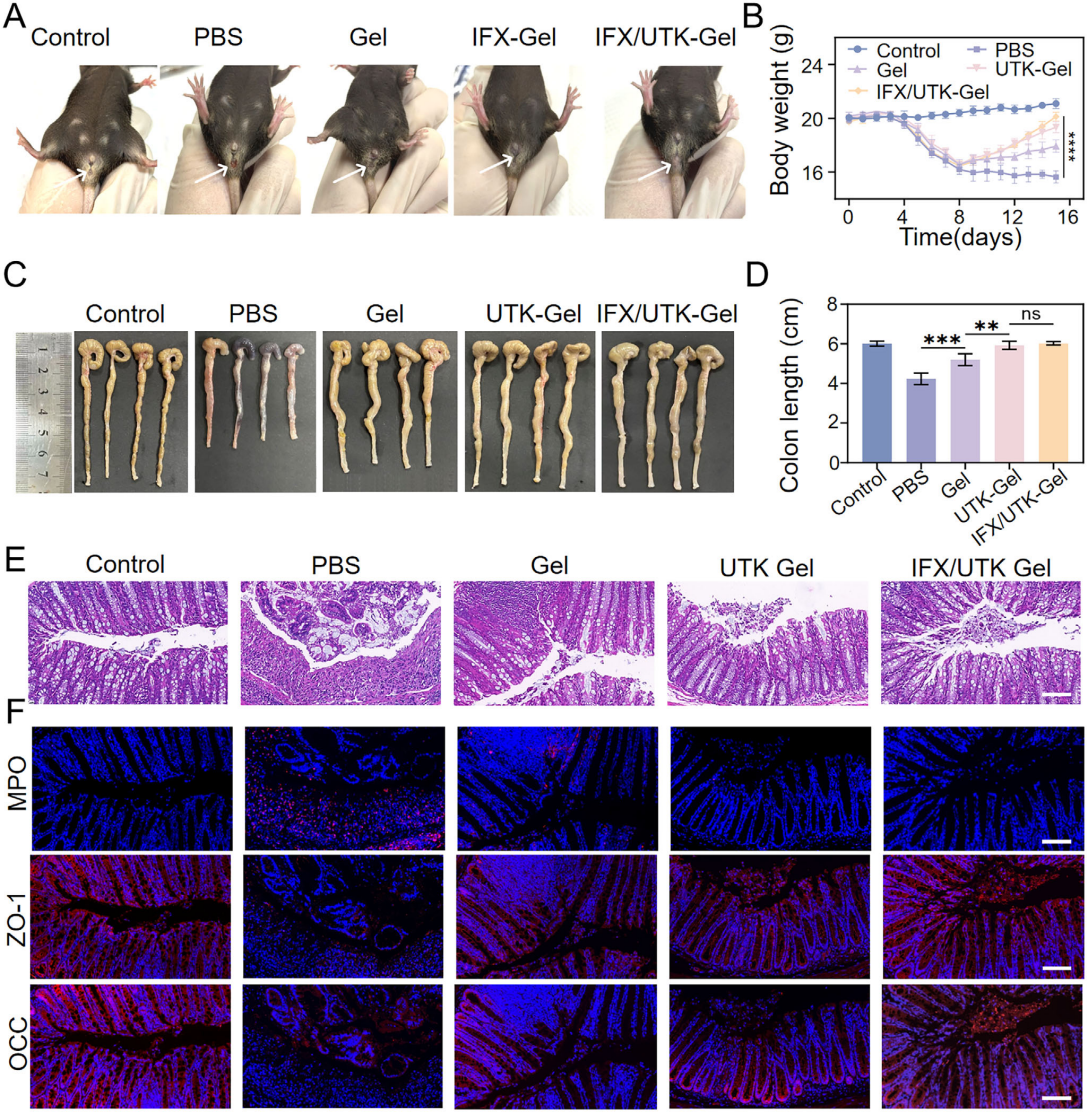
Therapeutic performance of UTK@predaGel and IFX/UTK@predaGel in a DSS‐induced colitis mouse model. (A) Representative photographs of rectal areas. (B) Body weight changes (*n* = 4). (C) Representative colon images and (D) colon length measurements (*n* = 4). (E) Representative H&E‐stained sections of colonic tissue. (F) Immunofluorescence analysis of MPO, ZO‐1, and occludin in colon tissues. Scale bars, 100 µm. (Data are expressed as mean ± SD; ^**^ *p* < 0.01, ^***^ *p* < 0.001, ^****^ *p* < 0.0001).

We further extended predaGel for oral delivery of ustekinumab (UTK), which has been approved for treating Crohn's disease and ulcerative colitis [51], and IFX/UTK, two antibodies (IFX/UTK‐Gel) in the DSS‐induced colitis model. Oral UTK‐Gel effectively alleviated disease progression, as evidenced by reduced anal bleeding (Figure 7A), steady weight recovery (Figure 7B), lower DAI scores (Figure S31), suppressed colon shortening (Figure 7C,D), and ameliorated splenomegaly (Figure S32). UTK‐Gel revealed significantly greater anti‐inflammatory effect than blank Gel, indicating efficient delivery of UTK to the inflamed colon. H&E staining confirmed that UTK‐Gel substantially reduced the depth of mucosal inflammatory infiltration and the degree of crypt architectural damage. Blinded histopathological scoring confirmed significantly lower inflammation scores in UTK‐Gel‐treated mice (Figure S33). Consistently, immunofluorescence staining for IL‐17A revealed a marked reduction of IL‐17A‐positive cells in the colonic mucosa of UTK‐Gel‐treated mice (Figure S34). Interestingly, IFX/UTK‐Gel demonstrated further better treatment effect, as characterized by lower MPO levels and more pronounced enhancement of intestinal barrier repair markers (ZO‐1 and occludin) than UTK‐Gel.

## 3 Conclusions

We have demonstrated that oral prebiotic polysaccharide hydrogels based on inulin and sodium alginate‐dopamine conjugate (predaGel), which mediate colon‐specific delivery of antibodies targeting TNF‐α and IL‐12/23, effectively scavenge ROS and restore gut microbiota. predaGel possesses desired properties for IBD therapy: (i) it can protect antibodies from gastric degradation while enable efficient antibody release at inflamed colon; (ii) its mucoadhesion and ROS scavenging ability not only significantly prolongs mucosal retention and antibody release but also improves anti‐inflammatory effects; (iii) prebiotic metabolites from inulin fermentation would promote beneficial SCFA‐producing flora and enhance Treg cell differentiation, which on one hand restores immune homeostasis and on the other hand prevents diarrhea. This oral antibody formulation is not only patient‐friendly but also offers an effective and well‐tolerated alternative to clinically applied i.v. injection. predaGel thus represents an emerging treatment strategy for IBD.

## 4 Experimental Section

### 4.1 The Preparation of IFX@predaGel

First, inulin was dissolved in deionized water at 80°C to a concentration of 0.8 g mL^−^^1^ and cooled to room temperature. Next, 500 µL of DA‐SA solution (0.1 g mL^−^^1^) was prepared and mixed with 500 µL of the cooled inulin solution under stirring to obtain a homogeneous blend. Subsequently, varying amounts of IFX and CaCl_2_ were added to the mixture, followed by incubation at 4°C overnight to form IFX@predaGel. The preparation of IFX@preGel was the same as that of IFX@predaGel, except that the DA‐SA solution was replaced with an SA solution.

### 4.2 Characterization

The morphology of the hydrogels was examined by scanning electron microscopy (SEM, Zeiss Supra 55). UV–vis absorption spectra were recorded on a Lambda 950 UV–vis spectrophotometer. Fourier transform infrared (FTIR) spectra were recorded on a Tensor 27 FT‐IR spectrometer (Bruker, Switzerland) with KBr pellets in the 500−4000 cm^−^^1^ region. ^1^H NMR spectra were acquired using a nuclear magnetic resonance spectrometer (Bruker, 400 MHz) with deuterium oxide (D_2_O) as the solvent. The molecular weight of sodium alginate was analyzed by gel permeation chromatography (GPC, PL‐GPC50) with an RI detector and two PLgel aquagel‐OH Mixed‐M columns (8 µm, 7.5 × 300 mm), using 0.1 m NaNO_3_ as the mobile phase at 1.0 mL min^−1^ and 40°C. All characterizations were carried out at room temperature.

### 4.3 Oxidative Stress Assay

For oxidative stress assessment, RAW264.7 cells were cultured in 24‐well plates containing cell slides at a density of 1.5 × 10^4^ cells per well. Once cells adhered, the culture medium was exchanged for high‐glucose DMEM containing LPS (100 ng mL^−^^1^) along with preGel, predaGel, or IFX@predaGel. Following 24 h of incubation, cells were rinsed with PBS and then labeled using the ROS‐sensitive dye DCFH‐DA. After three additional washes with PBS, intracellular ROS fluorescence was visualized using confocal laser scanning microscope (LSM880, Zeiss, Germany). For flow cytometric quantification of ROS levels (Cytoflex, Beckman, USA), DCFH‐DA‐stained cells received three washes with PBS, were then detached from the wells, and resuspended in 1 mL of PBS prior to analysis.

### 4.4 Anti‐Inflammatory Activity

RAW264.7 macrophages were placed in 24‐well plates that had been pre‐loaded with cell slides, at 1.5 × 10^4^ cells per well. Upon adherence, the culture medium was exchanged for high‐glucose DMEM containing lipopolysaccharide (LPS, 100 ng mL^−^^1^) along with preGel, predaGel, or IFX@predaGel. Following 24 h of treatment, the culture supernatants were collected. The levels of inflammatory cytokines, including TNF‐α, IL‐6, IL‐10, and TGF‐β, were quantified using ELISA kits (eBioscience) in accordance with the manufacturer's instructions.

### 4.5 The Therapeutic Effect of Hydrogel in DSS‐Induced UC Mouse Model

All animal procedures were approved by the Animal Care and Use Committee of Soochow University and carried out in compliance with the institutional guidelines for the care and use of laboratory animals (no. 202502A0832). A total of 28 Female C57BL/6 mice (6–8 weeks) were maintained under specific‐pathogen‐free (SPF) conditions and randomly assigned to seven groups. Mice in the control group received only sterile water, whereas acute colitis was induced in the other groups by providing DSS in drinking water ad libitum over a period of seven days. On day 8, colitic mice were allocated into six treatment groups and received one of the following interventions daily for seven consecutive days: PBS, predaGel alone (Gel), IFX@predaGel loaded with low‐ (5 mg kg^−1^), medium‐ (10 mg kg^−1^), or high‐dose (20 mg kg^−1^) IFX (denoted as IFX(L)‐Gel, IFX(M)‐Gel, and IFX(H)‐Gel, respectively), and a group receiving intravenous injection of IFX (5 mg kg^−1^, IFX‐iv). All formulations were administered in a 200 µL volume. During the treatment period, body weight, stool consistency, and fecal occult blood were recorded daily. The DAI was derived from the average of three subscores: percentage of body weight loss (scored 0–4), stool consistency (scored 0, 2, or 4), and rectal bleeding (scored 0, 2, or 4), using the formula DAI = (weight loss score + stool consistency score + bleeding score) / 3. At day 15, all mice were humanely sacrificed, after which spleens, blood, fecal samples, and colon tissues were harvested for subsequent analysis.

To evaluate UTK monotherapy and the combination of UTK with IFX, colitic mice were randomly divided into four groups (*n* = 5 per group) and treated daily for 7 days with PBS, Gel, UTK‐Gel (containing 5 mg kg^−1^ UTK), or IFX/UTK‐Gel (containing 10 mg kg^−1^ IFX and 5 mg kg^−1^ UTK). All formulations were administered in a 200 µL volume. All other procedures (DSS induction, DAI scoring, sample collection) were identical to those described above.

### 4.6 Flow Cytometry Analyses

Single‐cell suspensions prepared from the colonic lamina propria, the blood, spleen, and mesenteric lymph nodes of mice with colitis (after various treatments) were stained according to the manufacturer's protocols for flow cytometry analysis using the following antibodies: For Treg cell evaluation: anti‐CD45‐PE, anti‐CD4‐FITC, anti‐CD25‐PE/Cy5, and anti‐Foxp3‐APC. For TNF‐α‐producing cell evaluation: anti‐CD45‐FITC, anti‐CD11b‐PE/Cy7, and anti‐TNF‐α‐APC. For Th17 cell evaluation: anti‐CD45‐PE, anti‐CD4‐FITC, and anti‐IL‐17A‐APC. For transmembrane TNF (tmTNF)‐expressing cell evaluation: anti‐CD45‐FITC, anti‐CD11b‐PE/Cy7, anti‐F4/80‐PE, and anti‐transmembrane TNF (tmTNF)‐APC.

## Author Contributions

**Huan He**: investigation, methodology, writing – original draft, funding acquisition, conceptualization. **Zhiyuan Zhong**: writing – review and editing, methodology, project administration, funding acquisition, conceptualization. **Li Cao**: resources. **Xinyi Dong**: investigation, visualization, data curation. **Fenghua Meng**: resources, supervision.

## Conflicts of Interest

The authors declare no conflicts of interest.

## Ethics Approval Statement

Female C57BL/6 mice (6–8 weeks) were purchased from the Shanghai Laboratory Animal Center (Shanghai, China). All animal experiments were approved by the Animal Care and Use Committee of Soochow University, and all protocols conformed to the Guide for the Care and Use of Laboratory Animals (approval number: 202502A0832).

## Supporting information




**Supporting File**: advs76425‐sup‐0001‐SuppMat.docx.

## Data Availability

The data that support the findings of this study are available from the corresponding author upon reasonable request.
