# Supplementary material for: Oral Prebiotic Polysaccharide Hydrogels Sustaining Colon Antibody Release Alleviate Inflammatory Bowel Disease
Source: Adv Sci (Weinh). 2026 Jul 6;13(54):e76425. doi: 10.1002/advs.76425 (PMC13335760; doi:10.1002/advs.76425)
Supplement: Supplementary file 1 [file ADVS-13-e76425-s001.docx]

Supporting Information

Oral Prebiotic Polysaccharide Hydrogels Sustaining Colon Antibody Release Alleviate Inflammatory Bowel Disease

Xinyi Dong, Huan He*, Li Cao, Fenghua Meng, and Zhiyuan Zhong*

Xinyi Dong and Huan He contributed equally to this work.

**Supplementary Information contains:**

Experimental Section

Supplementary Figures S1-S29

**Experimental Section**

**Materials**

Dopamine hydrochloride (DA) was procured from Sigma-Aldrich St. Louis, MO, USA. Other reagents including Sodium alginate (SA, Mw 174 kDa), 1-Ethyl-3-(3-dimethylaminopropyl)carbodiimide hydrochloride (EDC), N-hydroxysuccinimide (NHS), and Calcium chloride (CaCl_2_) were procured from Rhawn, Shanghai, China. Inulin (In) was secured from Aladdin, Shanghai, China. Unless otherwise specified, all the aforementioned substances were utilized in their received state without further modification.

**The preparation of DA-SA**

The synthesis of catechol-modified sodium alginate (DA-SA) was executed via an EDC/NHS coupling reaction^[1]^. A solution was first prepared by dissolving 1.2g of SA in 400 mL of deionized water, which was later subjected to nitrogen purging for 30 min. The pH of this solution was manipulated to a reading of 5.5 by the addition of 1 M HCl. Subsequently, a mixture of 1.704 g EDC and 1.023 g NHS dissolved in a 16 mL 1:1 H_2_O: DMSO volume ratio was introduced. The synthesis progressed with the addition of 1.704 g of DA. The solution's pH was subsequently maintained 5.5 over a duration of 12 hours. The DA-SA solution then underwent dialysis (Mw cutoff: 10000 Da) against a pH 5.5 aqueous solution, periodically adjusted with 1 M HCl, over a period of 2 days to effectively remove the residual reactants and salt. Another round of dialysis was performed against deionized water for an additional day to ensure complete removal of unreacted reagents and salts.

**Adhesion properties of the hydrogels**

The adhesive strength between the hydrogel with different concentrations of CaCl_2_ (24 mm × 24 mm) and the rabbit colon substrate was tested using a universal testing machine (5567, Instron, USA) at a constant speed of 5 mm s^-1^. The adhesive strength P (kPa) between the hydrogel and the substrate was calculated using the equation:

*P=F/S* (Equation S1)

where, *F* (N) is the maximum tensile adhesion between the hydrogel and the substrate, and *S* (mm^2^) is the sample area.

**Compressive properties of the hydrogels**

The compression performance of the hydrogel with different concentration of CaCl_2_ was tested by a universal testing machine (UTM, Instron 5567, USA) with a 100 N force sensor and a loading rate of 5 mm min^-1^. The cylindrical hydrogel used for the compression test has a height of 10 mm and a diameter of 8 mm. The calculation formula for compressive strength (S) is as follows:

*S=Q/A* (Equation S2)

*Q* (N) is the compression load, *A* (mm^2^) is the cross sectional area of the hydrogel.

**IFX release behavior**

We immersed Cy5.5-IFX@predaGel, with a diameter of 8 mm and a height of 1mm, into 3 mL of PBS at a temperature of 37 °C. At specified time intervals, we withdrew 1.5 mL of the solution, compensating with an equivalent volume of fresh PBS. We established the incubation solution from predaGel as the baseline. Consequently, we examined the absorbance of Cy5.5-IFX in Cy5.5-IFX@predaGel incubation solutions using a UV-Vis spectrometer.

**Stability of IFX@predaGel in simulated gastrointestinal fluid**

The stability of the IFX@predaGel in simulated gastrointestinal fluid was evaluated as previously described, with modifications^[2]^. Firstly, IFX@predaGel was prepared by mixing 1 μL Cy5.5-IFX (0.1 g mL⁻¹), 500 μL DA-SA solution (0.1 g mL⁻¹), and 500 μL inulin solution (0.8 g mL⁻¹). Then, 2 mL of simulated gastric fluid (SGF, pH = 2, containing 10 mg mL⁻¹ pepsin) was added to a petri dish containing the freshly prepared IFX@predaGel, followed by incubation for 2 h at 37 °C. After removing the SGF, 2 mL of simulated intestinal fluid (SIF, pH = 6.8) was added to the petri dish, followed by incubation at 37 °C for 4 h. After removing the SIF, 2 mL of simulated colonic fluid (SCF, pH 7.8) was added to the petri dish, followed by incubation at 37 °C for a specified duration.

The IFX@predaGel was removed from the simulated solution and washed with DI water for 2-3 times. The remaining hydrogel was weighed after drying. The degradation rate of the hydrogel at different stages was calculated as follows:

*Degradation rate= (initial weight − remaining weight)/initial weight* (Equation S3)

The release rate of Cy5.5-IFX in the hydrogel was obtained by monitoring the ultraviolet absorption of Cy5.5-IFX at 680 nm in the simulated liquid.

**Gastrointestinal stability and colonic inulinase-responsive degradation of IFX@predaGel**

To avoid autofluorescence from different tissues, the IFX were labeled with the near-infrared fluorescent dye Cy5.5 to evaluate the stability of the Cy5.5-IFX after oral administration. After 4 days of feeding with 3% dextran sodium sulfate (DSS, Millipore), Cy5.5-IFX (200 μL, 0.5 mg mL^-1^), Cy5.5-IFX@preGel (200 μL, Cy5.5-IFX: 0.5 wt.‰), and Cy5.5-IFX@predaGel (200 μL, Cy5.5-IFX: 0.5 wt.‰) were orally administered to the mice after fasting for 48 h. At different time periods (2, 6, 12, and 24 h), the abdominal fluorescence in the mice was recorded using an IVIS small animal fluorescence imaging system (Lumina III). The mice were sacrificed 24 h after hydrogels gavage, and the removed colon was imaged using IVIS small animal fluorescence imaging to observe the retention of the Cy5.5-IFX in the colon.

**Cytotoxicity evaluation of IFX@predaGel**

The cytotoxicity of the IFX@predaGel against RAW264.7 cells was assessed using the 3-(4,5-dimethylthiazol-2-yl)-2,5-diphenyltetrazolium bromide (MTT) assay from Solarbio. The RAW264.7 cells were cultured in Dulbecco's modified Eagle's medium (DMEM; HyClone, USA), augmented with 10% fetal bovine serum (HyClone) and 1% penicillin-streptomycin solution (HyClone), in a CO_2_ incubator maintained at 37 °C. To begin, 500 μL of RAW264.7 cells (1×10^5^ cells mL^-1^ in DMEM) were added to each well and incubated in a CO_2_-enriched atmosphere (5%) at a temperature of 37 °C. Subsequently, the hydrogels with different IFX concentrations (0-2.0 wt.‰) were introduced into 48-well plate. Following a 24-hour incubation period, 100 μL of 5 mg mL^-1^ MTT solution was added to each well and incubated at 37 °C for 4 h. Post incubation, the medium was removed and 200 μL of dimethyl sulfoxide was added to each well, incubating the samples for an additional 20 minutes. Lastly, the absorbance of the medium was measured at 490 nm using an enzyme-linked immunosorbent assay (ELISA) microplate reader. Cell viability was expressed as a relative percentage of absorbance in comparison with that of the control, which did not contain any hydrogel.

**Animal and DSS-induced mouse models**

C57BL/6 mice (female, 8 weeks old) were obtained from Beijing Weitonglihua Laboratory Animal Technology Co., Ltd. All animal experiments were approved by the Animal Care and Use Committee of Soochow University and conducted according to the Guidelines for the Care and Use of Experimental Animals.

**Histological scoring**

Colonic tissue sections (H&E stained) were evaluated blindly by two independent observers. The following parameters were scored: (i) inflammatory cell infiltration (0–4), (ii) crypt damage (0–4), and (iii) mucosal erosion (0–4), according to previously described criteria ^[3]^. A total histological score (0–12) was calculated by adding the three individual scores.

**Quantitative real-time PCR**

Cells were lysed in TRIzol (Thermo Fisher), and total RNA was extracted via chloroform-isopropanol precipitation following the manufacturer’s protocol. RNA was reverse-transcribed into cDNA using PrimeScript™ RT Master Mix (Takara). Quantitative real-time polymerase chain reaction (qRT-PCR) was performed with SYBR Green PCR Mix (Toyobo) on an FTC-3000 system. Gene expression levels of IL-10, IL-6, TNF-α, and TGF-β were quantified using the following primers: interleukin-10 (IL-10) (forward, AATAAGCTCCAAGACCAAGGTGT; reverse, CATCATGTATGCTTCTATGCAGTTG), IL-6 (forward, ACTTCCATCCAGTTGCCTTCTTGG; reverse, TTAAGCCTCCGACTTGTGAAGTGG), TNF-α (forward, GATGGGTTGTACCTTGTCTACT; reverse, CTTTCTCCTGGTATGAGATAGC), and TGF-β (forward, CCAGATCCTGTCCAAACTAAGG; reverse, CTCTTTAGCATAGTAGTCCGCT). Data were normalized to GAPDH and analyzed using 2^–ΔΔCT^ method.

**Quantification of inflammatory cytokines in blood**

Serum samples were isolated from mice after various treatments and diluted for analysis. The levels of TNF-α and IL-6 in the serum were measured by ELISA (eBioscience).

**Supplementary Figures S1-S29**

**Figure S1.** (A) Proposed reaction scheme of DA-SA conjugate. (B) Typical ^1^H NMR spectra of DA-SA. (C) UV-Vis of SA and DA-SA. (D) FTIR spectra of SA and DA-SA.

**Figure S2.** Injectable property of IFX@predaGel.

**Figure S3.** Adhesion of IFX@predaGel to different tissues.

**Figure S4.** The IFX@preGel adhered to rabbit colon fell off during complete immersion in PBS for 5 min.

**Figure S5.** Compressive strength of the IFX@predaGel with varying concentrations of CaCl_2_.

**Figure S6.** The swelling property of the IFX@predaGel with varying concentrations of CaCl_2_.

**Figure S7.** Compressive strength of the IFX@predaGel with inulin-to-DA-SA mass ratio increased from 0:1 to 16:1.

**Figure S8.** The anti-swelling property of the IFX@predaGel with inulin-to-DA-SA mass ratio increased from 0:1 to 12:1.

**Figure S9.** Inulin gel failed to maintain structural integrity and disintegrated within 1 min in PBS.

**Figure S10.** Release rate of Cy5.5-IFX in Cy5.5-IFX@predaGel, Cy5.5-IFX@Ca^2+^/DA-SA Gel (without inulin).

**Figure S11. TNF-α levels after 24-h co-culture of LPS-stimulated macrophages with IFX@predaGel.**

**Figure S12.** Stability of IFX@predaGel after storage at 4 °C for 0, 7, and 14 days. (A) Morphology of the IFX@predaGel. (B) In vivo fluorescence imaging and (C) quantitative analysis of Cy5.5-IFX@predaGel; the stable fluorescence intensity indicates that the encapsulation efficiency of IFX remained unchanged during storage (n = 6). (D) TNF‑α levels in supernatants after 24 h of co‑culture of LPS‑stimulated macrophages with IFX@predaGel stored for the indicated times (n = 3).

**Figure S13.** DPPH• scavenging ratios were measured for preGel (without dopamine) and predaGel.

**Figure S14.** Cytotoxicity of IFX@predaGel with different IFX concentrations.

**Figure S15.** Images of IFX@predaGel after 2 h in SGF, 4 h in SIF, 4 h and 12 h in SCF (SGF+SIF+SCF), or after 2 h in SGF, 4 h in SIF, 4 h and 12 h in SCF plus inulinase (SGF+SIF+SCF(Inulinase)).

**Figure S16.** Real-time monitoring images of IFX@predaGel obtained by an in vitro simulated human gastric system.

**Figure S17.** **TNF-α levels in LPS-stimulated macrophages after 24-h treatment with SGF/SIF-exposed IFX@predaGel.**

**Figure S18.** The adhesive strength of IFX@predaGel after incubation in SGF for 2 h, SIF for 4 h, and SCF for 4 h.

**Figure S19.** Representative photographs of rectal areas of a healthy mouse (Control) and colitis mice after therapy of Gel, IFX(L)-Gel, IFX(M)-Gel, IFX(H)-Gel, or IFX-iv (on the 15 day).

**Figure S20.** Spleen weight of healthy mouse (Control) and colitis mice after therapy of Gel, IFX(L)-Gel, IFX(M)-Gel, IFX(H)-Gel, or IFX-iv (on the 15 day). n =4.

**Figure S21.** Histological inflammation scores of colonic tissue sections (quantification of Figure 4F).

**Figure S22.** Immunohistochemical staining of MPO in colon tissues.

**Figure S23.** Relative abundance of *Desulfovibrionales* at the order level in the microbial community.

**Figure S24.** Relative abundance of *Ruminococcaceae* at the family level in the microbial community based on heatmap analysis.

**Figure S25. (A) The representative flow dots** and (B) percentages **of Treg (CD45^+^CD4^+^CD25^+^Foxp3^+^) in the peripheral blood.**

**Figure S26.** Representative flow cytometry analyses and percentages of **CD4**^+^ cells in **CD45**^+^ **cells** **in** colonic lamina propria (LP).

**Figure S27.** Representative flow cytometry analyses of TNF-α in CD11b^+^ cells in **peripheral blood.**

**Figure S28.** Serum concentrations of TNF-α and IL-6 of healthy mouse (Control) and colitis mice after therapy of IFX(M)-Gel and IFX-iv on days 8-14. n = 3.

**Figure S29**. IFX(M)-Gel administration reduces inflammation in the colon. (A) Representative flow cytometry analyses of mTNF⁺ cells in CD11b^+^ cells in **colonic lamina propria. (B, C)** Representative flow cytometry analyses and percentages of mTNF⁺ cells in CD11b^+^ cells in **peripheral blood**. (D, E) Representative flow cytometry analyses and percentages of IL-17A expression in mesenteric lymph nodes (MLN). n = 3.

**Figure S30.** (A) H&E staining images of heart, liver, spleen, lung, and kidney tissues of mice after gavage administration of IFX(M)-Gel for 7 d. (B) H&E staining images of colon tissues of mice after gavage administration of IFX(M)-Gel for 7 d. (C) Body weight changes (n=3).

**Figure S31.** DAI scores of healthy mouse (Control) and colitis mice after therapy of Gel, UTK-Gel, or IFX/UTK-Gel. n =4.

**Figure S32.** Spleen weight of healthy mouse (Control) and colitis mice after therapy of Gel, UTK-Gel, or IFX/UTK-Gel (on the 15 day). n =4.

**Figure S33.** Histological inflammation scores of colonic tissue sections (quantification of Figure 7E).

**Figure S34.** Immunofluorescence staining of IL-17A in colon tissues.

**Reference**

[1] J. Lee, K. Chang, S. Kim, V. Gite, H. Chung, D. Sohn, *Macromolecules* **2016**, *49*, 7450-7459.

[2] A. Brodkorb, L. Egger, M. Alminger, P. Alvito, R. Assuncao, S. Ballance, T. Bohn, C. Bourlieu-Lacanal, R. Boutrou, F. Carriere, A. Clemente, M. Corredig, D. Dupont, C. Dufour, C. Edwards, M. Golding, S. Karakaya, B. Kirkhus, S. Le Feunteun, U. Lesmes, A. Macierzanka, A. R. Mackie, C. Martins, S. Marze, D. J. McClements, O. Menard, M. Minekus, R. Portmann, C. N. Santos, I. Souchon, R. P. Singh, G. E. Vegarud, M. S. J. Wickham, W. Weitschies, I. Recio, *Nat. Protoc.* **2019**, *14*, 991.

[3] S. Wirtz, V. Popp, M. Kindermann, K. Gerlach, B. Weigmann, S. Fichtner-Feigl, M. Neurath, *Nat. Protoc.* **2017**, *12*, 1295–1309.
